# ZmWRKY104–ZmCCaMK interaction enhances brassinosteroid-promoted salt tolerance in maize (*Zea mays* L.) via antioxidant defense

**DOI:** 10.1080/15592324.2025.2611639

**Published:** 2026-01-03

**Authors:** Li-li Zhao

**Affiliations:** a Taiyuan Ecological Environment Publicity and Education Center, Taiyuan Shanxi Province, People's Republic of China

**Keywords:** ZmWRKY104, ZmCCaMK, brassinosteroid, salt tolerance, antioxidant defense, maize

## Abstract

Brassinosteroid (BR)-mediated salt tolerance is a crucial mechanism for maize (*Zea* *mays* L.) adaptation to saline-alkaline environments. This study aimed to elucidate the molecular mechanism underlying BR-induced salt tolerance in maize, focusing on the regulatory roles of ZmWRKY104 and ZmCCaMK. Key results showed that ZmWRKY104 directly interacts with ZmCCaMK in the nucleus in a non-phosphorylation-dependent manner, forming a novel regulatory module. BR treatment upregulates *ZmWRKY104* expression, and overexpression of *ZmWRKY104* significantly enhances the activities of antioxidant enzymes (APX and SOD). Co-expression of *ZmWRKY104* and *ZmCCaMK* synergistically promotes the antioxidant defense system in maize. Transgenic maize overexpressing *ZmWRKY104* exhibits obvious salt tolerance advantages under 100 mM NaCl stress compared to wild-type plants, including reduced leaf yellowing, increased plant height and root length, as well as decreased electrolyte leakage (EL) and malondialdehyde (MDA) content. Collectively, this study identifies a novel non-phosphorylation-dependent WRKY-CCaMK regulatory module in the BR signaling pathway, which enhances BR-induced maize salt tolerance by synergistically activating antioxidant defense. The findings highlight *ZmWRKY104* as a candidate gene and provide a potential molecular mechanism for salt-tolerant maize breeding in saline-alkaline regions of northern China.

## 1 Introduction

Saline-alkaline land covers ~100 million hectares in China, with 7.6 million hectares of cultivated land affected by salinization, primarily in arid/semi-arid regions north of the Huaihe River, northwest China, and Xinjiang.^1^^,^^2^ Salt stress induces oxidative damage in maize via osmotic pressure and ion toxicity, reducing yields by 20–30%.^3^^,^^4^ Cultivating salt-tolerant varieties is critical for sustainable maize production in these regions.

Brassinosteroids (BRs) are key hormones regulating plant stress responses, improving abiotic tolerance by activating antioxidant systems.^1^^,^^5^ Calcium/calmodulin-dependent protein kinase (CCaMK) acts as a central signaling hub in abiotic stress,^6^^,^^7^ with synergistic regulation with BR emerging as a research focus.^1^^,^^5^ WRKY transcription factors are well-documented in plant stress defense,^8‐16^ but their interaction with CCaMK in BR-induced salt tolerance remains unreported in maize.

This study identified ZmWRKY104 as a ZmCCaMK-interacting protein, investigating its role in BR-mediated antioxidant defense and salt tolerance. The findings provide new insights into plant salt stress responses and valuable genetic resources for maize improvement in saline-alkaline regions.

## 2 Materials and methods

### 2.1 Plant materials and treatments

Maize (*Zea mays* L. cv. B73) and *Nicotiana benthamiana* seeds were maintained by the laboratory. Two-week-old maize seedlings were treated with 50 nmol/L BR (oleuropein lactone, Sigma-Aldrich, USA) or 100 mM NaCl (Solarbio, China) for 0, 15, 30, 45, 60, 90, 120, or 240 min; controls were treated with distilled water. The second leaf was harvested, frozen in liquid nitrogen, and stored at −80 °C.^17^

### 2.2 Protein interaction assays

**GST-pull down:** Purified GST-ZmWRKY104 and His-ZmCCaMK proteins (expressed in E. *coli* BL21) were incubated with GST affinity resin (GE Healthcare, USA) at 4 °C for 2 h. Bound proteins were detected by Western blotting using anti-His (Abcam, ab18184) and anti-GST (Abcam, ab19256) antibodies.^17^

**LCI assay:** ZmWRKY104 was cloned into pC1300-nLUC, and ZmCCaMK into pC1300-cLUC. The constructs were co-transformed into *N*. *benthamiana* leaves via Agrobacterium GV3101. Luciferase signals were detected using an in vivo imaging system (Tanon-5200, China) after 48 h.^18^

**BiFC assay:** ZmWRKY104 (CDS without terminator) was cloned into pSPYNE (*YFP*^*N*^-*ZmWRKY104*), and ZmCCaMK into pSPYCE (*YFP*^*C*^-*ZmCCaMK*). The vectors were co-infiltrated into 4-week-old *N*. *benthamiana* leaves, and YFP signals were observed via confocal microscope (Zeiss LSM710, Germany).^17^^,^^19^

### 2.3 *In Vitro* kinase assay

The reaction system (50 μL) contained 25 mM Tris-HCl (pH 7.5), 5 mM MgCl₂, 1 mM DTT, 2.5 mM CaCl₂, 2 μM calmodulin (CaM), 200 nmol/L ATP, 10 μCi [*γ*- ³²P] ATP, 0.01 mg/mL ZmCCaMK, and 1 μg His-ZmWRKY104 (or MBP as positive control). After incubation at 30 °C for 30 min, products were separated by 12% SDS-PAGE and detected by radioautography.^17^

### 2.4 Vector construction

The *pXZP008* vector, which harbors the *mCherry* gene driven by the ubiquitin promoter, was used for protoplast transient expression assays as described previously.^17^ The coding region of *ZmWRKY104* was amplified by PCR and cloned into the *pXZP008* vector using *BamHI* and *KpnI* restriction enzymes, generating the *ubi:ZmWRKY104-mCherry* plasmid. To construct the dominant-negative *ubi:ZmWRKY104-SRDX-mCherry* plasmid, the exogenous EAR motif repression domain SRDX (5’-CTGGATCTAGAACTCCGTTTG-3’) was introduced into the C-terminus of *ZmWRKY104* via PCR, following the method described by.^20^

### 2.5 Antioxidant enzyme activity detection

Maize protoplasts were isolated from B73 leaves (Sheen laboratory protocol, modified). Protoplasts were transfected with *ZmWRKY104* overexpression (pXZP008-*ZmWRKY104*)/SRDX vector (pXZP008-*ZmWRKY104-SRDX*) or dsRNA (for silencing) via PEG-mediated method. After 24 h, APX and SOD activities were measured using kits (Solarbio, China).^5,19‐24^

### 2.6 Transgenic maize salt tolerance evaluation

*ZmWRKY104* overexpression lines (#15, #17) and wild-type (WT) maize were grown in 1/2 Hoagland solution. At the 3-leaf stage, seedlings were treated with 100 mM NaCl for 4 days. Phenotypes (leaf yellowing, plant height, root length) were recorded, and electrolyte leakage (EL) and malondialdehyde (MDA) content were measured.^17^

### 2.7 Statistical analysis

Data were analyzed using GraphPad Prism 8.2.1. Significant differences were determined by Duncan’s multiple comparisons (*p* < 0.05). All experiments had ≥3 biological replicates.

## 3 Results and discussion

### 3.1 ZmWRKY104 directly interacts with ZmCCaMK *in* *Vitro* and *in* *Vivo*

**Figure 1.**
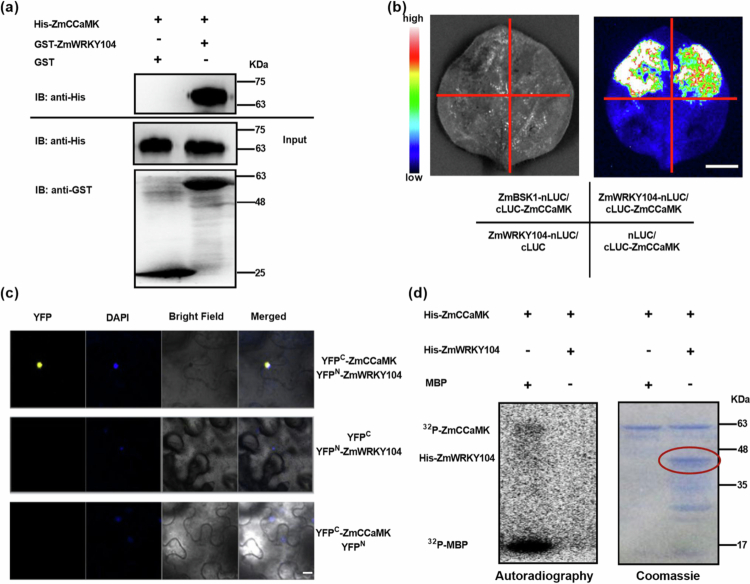
ZmWRKY104 directly interacts with ZmCCaMK in a non-phosphorylation-dependent manner. (a) GST-pull down assay showing binding between GST-ZmWRKY104 and His-ZmCCaMK. GST/His-ZmCCaMK was used as a negative control. (b) LCI assay showing strong luciferase signals in co-transformed *N*. *benthamiana* leaves. ZmBSK1-nLUC/cLUC-ZmCCaMK was used as a positive control, and ZmWRKY104-nLUC/cLUC and nLUC/cLUC-ZmCCaMK were used as negative controls. Scale bar, 1 cm. (c) BiFC assay showing nuclear YFP signals in co-expressing leaf cells (DAPI = nuclear stain). Scale bar, 20 µm. (d) *In vitro* kinase assay showing no ³²P-labeled ZmWRKY104 (MBP = positive control). The ZmWRKY104 band is located within the red ellipse. Data are means ± SE (*n* = 3, *p* < 0.05).

GST-pull down assay showed GST-ZmWRKY104 bound to His-ZmCCaMK, while the GST control did not (Figure 1a). LCI assay revealed strong luciferase signals in *N*. *benthamiana* leaves co-transformed with *ZmWRKY104*-nLUC +cLUC-*ZmCCaMK*, significantly higher than negative controls (Figure 1b). BiFC assay detected YFP signals in the nucleus of leaf cells co-expressing YFP^C^-*ZmCCaMK* and YFP^N^-*ZmWRKY104* (Figure 1c). These results confirm a direct nuclear interaction between ZmWRKY104 and ZmCCaMK— the first report of WRKY-CCaMK crosstalk in maize BR signaling. This nuclear localization aligns with the transcriptional regulatory role of WRKY proteins and the signaling function of CCaMK, suggesting they may co-regulate downstream gene expression.^7^^,^^25^

### 3.2 ZmCCaMK does not phosphorylate ZmWRKY104

CCaMK was initially identified as a key factor in root nodule symbiosis of legumes and arbuscular mycorrhizal symbiosis of mycorrhizal plants, and its role in stress response was subsequently confirmed. Previous studies have reported that CCaMK can phosphorylate its target factor CYCLOPS; however, the present study found a novel interaction between ZmCCaMK and ZmWRKY104 that is independent of phosphorylation.

*In vitro* kinase assays showed a ³²P-labeled band for MBP (positive control) but no ³²P-labeled band for ZmWRKY104 (Figure 1d), indicating that ZmWRKY104 is not a phosphorylation substrate of ZmCCaMK.

To highlight the innovation of this finding, we compared the interaction mode of CCaMK/CYCLOPS with that of ZmCCaMK/ZmWRKY104 based on relevant literature^26^^,^ ^27^), with the following differences: 1) Binding mode: The interaction between CCaMK and CYCLOPS is phosphorylation-dependent (CCaMK phosphorylates the Ser/Thr sites of CYCLOPS), which is a classic mechanism of root nodule symbiotic signal transduction in legumes. In contrast, the interaction between ZmCCaMK and ZmWRKY104 in this study is phosphorylation-independent (no phosphorylation band of ZmWRKY104 was detected in *in* *vitro* kinase assay), representing a novel non-phosphorylation-dependent interaction. 2) Functional localization: CCaMK/CYCLOPS mainly regulates the development of symbiotic organs, while ZmCCaMK/ZmWRKY104 focuses on the regulation of antioxidant defense under abiotic stress (salt stress). 3) Action mode: CCaMK activates the transcriptional function of CYCLOPS through phosphorylation, whereas ZmCCaMK may act as a “scaffold protein” to recruit ZmWRKY104 to the promoter regions of APX and SOD genes, and can activate downstream genes without phosphorylation. This comparison clearly highlights the innovative discovery of this study in the functional diversity of CCaMK.

Additionally,^1^ focused on drought stress and found that the autophosphorylation sites (Thr420 and Ser454) of ZmCCaMK are the key to BR-induced antioxidant defense. In contrast, the present study targets salt stress and reveals that ZmCCaMK regulates antioxidant defense through non-phosphorylation interaction with ZmWRKY104. This indicates that ZmCCaMK adopts a differentiated regulatory mechanism under different stress conditions: it directly activates the downstream pathway through autophosphorylation under drought stress, and regulates the pathway by forming a complex through protein interaction under salt stress, reflecting the diversity and specificity of the plant stress signal network.

### 3.3 *ZmWRKY104* positively regulates BR-induced antioxidant defense

**Figure 2.**
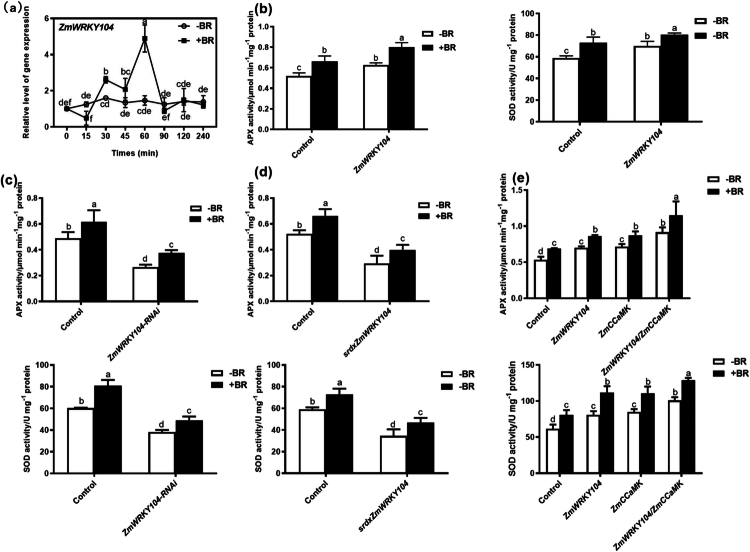
*ZmWRKY104* regulates BR-induced antioxidant defense, synergistically enhanced by *ZmCCaMK*. (a) qRT-PCR showing *ZmWRKY104* expression under BR treatment. White circles represent distilled water treatment, and black squares represent 50 nmol/L BR treatment. (b) Transient overexpression of *ZmWRKY104* increases APX/SOD activities. (c-d) Silencing/SRDX *ZmWRKY104* reduces basal antioxidant enzyme activities, which BR cannot restore. (e) Co-expression of *ZmWRKY104* and *ZmCCaMK* synergistically enhances APX/SOD activities. Different lowercase letters indicate significant differences (*n* = 3, *p* < 0.05).

qRT-PCR showed *ZmWRKY104* expression was upregulated by BR treatment, peaking at 60 min (4.8-fold vs. 0 h, Figure 2a), indicating *ZmWRKY104* is a BR-responsive gene. Transient overexpression of *ZmWRKY104* in protoplasts increased APX (20%) and SOD (19%) activities, with BR treatment further enhancing these activities (APX: +28%, SOD: +15%, Figure 2b). Silencing *ZmWRKY104* via dsRNA or *srdxZmWRKY104* reduced basal APX/SOD activities by 36–45%, and BR could not restore these activities (Figure 2c-d). These results demonstrate *ZmWRKY104* is essential for BR-induced antioxidant defense— consistent with BR’s role in activating antioxidant systems to mitigate salt-induced oxidative damage.^1^^,^^5^

### 3.4 *ZmWRKY104* and *ZmCCaMK* synergistically enhance BR-induced antioxidant defense

Co-expression of *ZmWRKY104* and *ZmCCaMK* in protoplasts increased APX (71%) and SOD (63%) activities— higher than single-gene expression (*ZmWRKY104* alone: +31% APX, +31% SOD; *ZmCCaMK* alone: +34% APX, +37% SOD, Figure 2e). BR treatment further elevated enzyme activities in the co-expression group (APX: +25%, SOD: +38%). This synergism suggests ZmCCaMK interacts with ZmWRKY104 to amplify BR-mediated antioxidant responses, potentially by stabilizing ZmWRKY104 or facilitating its binding to target gene promoters. The findings highlight the importance of the ZmWRKY104-ZmCCaMK module in fine-tuning BR-induced stress defense, which is critical for maize adapting to salt stress.

### 3.5 *ZmWRKY104* overexpression enhances maize salt tolerance

**Figure 3.**
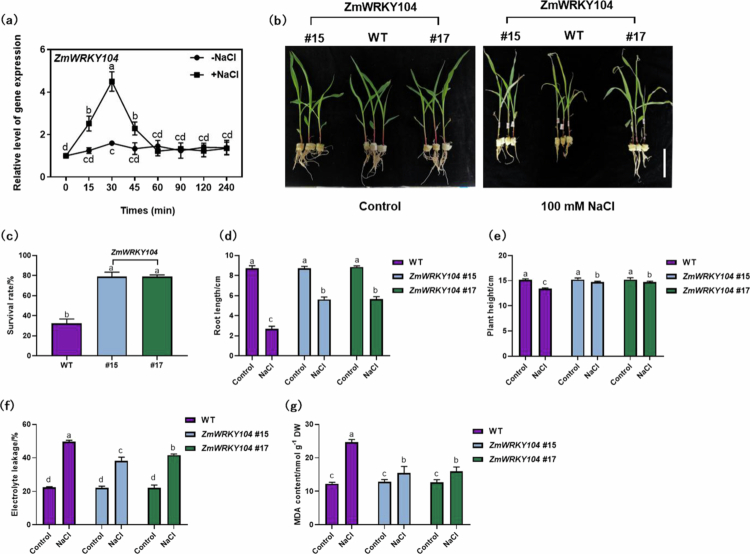
*ZmWRKY104* overexpression enhances maize salt tolerance. (a) qRT-PCR showing *ZmWRKY104* expression under NaCl treatment. (b) Phenotypes of *WT* and overexpression lines (#15, #17) under salt stress. Maize seedlings were treated with 100 mM NaCl for 4 days, with ~30 transgenic plants per replicate; representative images were captured at 4 days post-treatment. Scale bar, 5 cm. (c–e) Survival rate (Maize seedlings were treated with 100 mM NaCl for 7 d, ~30 plants per replicate), root length, and plant height of maize seedlings. (f-g) EL and MDA content in leaves under salt stress. Different lowercase letters indicate significant differences (*n* = 3, *p* < 0.05).

NaCl treatment upregulated *ZmWRKY104* expression (2.8-fold at 30 min, Figure 3a), indicating its involvement in salt stress responses. Under 100 mM NaCl, WT maize showed severe leaf yellowing (60–70% yellow leaves), reduced plant height (11% vs. control), and low survival rate (32%); overexpression lines (#15, #17) had less yellowing (20–25%), higher plant height (97% vs. control), and survival rate (79%, Figure 3b–e). Additionally, EL (40% vs. 50% in WT) and MDA (16 nmol/g FW vs. 25 nmol/g FW in WT) were lower in overexpression lines (Figure 3f-g). EL and MDA are indicators of membrane damage caused by oxidative stress, so their reduction confirms *ZmWRKY104* enhances salt tolerance by mitigating oxidative damage via antioxidant defense activation.

This practical significance is particularly relevant for northern China’s saline-alkaline regions, where maize yield is constrained by 100–150 mM NaCl. Introducing *ZmWRKY104* into local varieties (e.g., “Zhengdan 958”) via CRISPR/Cas9 could increase yield, addressing the regional challenge of saline-alkaline land utilization.

## 4 Conclusions

1. ZmWRKY104 directly interacts with ZmCCaMK in the nucleus via a non-phosphorylation-dependent mechanism.
2. *ZmWRKY104* positively regulates BR-induced antioxidant defense (APX/SOD activities), with synergistic enhancement by ZmCCaMK.
3. Overexpression of *ZmWRKY104* improves maize salt tolerance by reducing oxidative damage (lower EL and MDA).

*ZmWRKY104* is a promising candidate gene for salt-tolerant maize breeding, providing a practical strategy for sustainable agriculture in saline-alkaline regions.
